# Impact of Education and Gender on Knowledge, Attitudes, and Practices Toward Vitamin D Among the Lebanese Population With Emphasis on Young Adults

**DOI:** 10.7759/cureus.81861

**Published:** 2025-04-07

**Authors:** Suzana Salhab, Ibrahim A Srour, Imtithal Sheet, Lamis Karaki, Hadi Haddad, Mohamad Nour Zeineddine, Samer Sakr

**Affiliations:** 1 Department of Biological Sciences, Faculty of Science, Beirut Arab University, Beirut, LBN; 2 Department of Emergency Medicine, Lebanese University Faculty of Medicine, Beirut, LBN; 3 Department of Emergency, Al-Zahraa Hospital University Medical Center, Beirut, LBN; 4 Department of Biological and Chemical Sciences, Lebanese International University, Beirut, LBN; 5 Pediatric Department of Medicine, Lebanese American University Medical Center-Rizk Hospital, Beirut, LBN

**Keywords:** attitudes, health knowledge, lebanon, practice, vitamin d

## Abstract

Background

Vitamin D deficiency is a growing global health concern that affects populations, even in sun-rich regions such as Lebanon. Despite abundant sunlight, limited research has explored the knowledge, attitudes, and practices (KAP) related to vitamin D among young Lebanese adults.

Study design

This study employed an online quantitative cross-sectional design.

Methods

A self-administered online questionnaire distributed over six months (from August 2023 to January 2024) collected data on the participants' demographic characteristics, as well as their knowledge, attitudes, and practices related to vitamin D.

Results

Most participants (75.7%) had sufficient knowledge about vitamin D sources. The internet and physicians emerged as the dominant sources, cited by 175 participants, which represented 17.6% of the total responses related to vitamin D knowledge sources. Sunlight was perceived by the participants as the most crucial source. Additionally, 244 (33.7%) of the participants acknowledged the role of vitamin D in preventing osteoporosis, whereas 144 (20.4%) believed that it was aiding in calcium absorption. Significant levels of knowledge (defined as scores of ≥21 out of 40) and positive attitudes (defined as ≥4 correct answers out of six) were evident among more than 255 (75%) of the participants. However, over half expressed concerns about their vitamin D levels. Most were willing to take supplements (330, 92.7%) and undergo testing (326, 92.1%).

Conclusion

Although young Lebanese adults demonstrated good overall knowledge and positive attitudes toward vitamin D deficiency, implementing comprehensive awareness campaigns and health programs that emphasize behavior change is crucial for combating deficiency. These initiatives should focus on promoting tangible changes in practices, such as safe sun exposure, the consumption of vitamin D-rich foods, and adherence to testing and supplementation when necessary.

## Introduction

Vitamin D, popularly known as the sunshine vitamin, is an essential fat-soluble nutrient that plays a crucial role in overall good health. It is most well-known for assisting in promoting bone health by facilitating the absorption of phosphorus and calcium [1]. Vitamin D also contributes to immune system support, cell development regulation, and inflammation reduction [2]. An increased risk of many different ailments, including osteoporosis, diabetes, cardiovascular diseases, cancer, musculoskeletal disorders, and hypertension, has been associated with an insufficient amount of vitamin D [3].

Vitamin D is derived mostly from sun exposure, which is predominantly synthesized in the skin by activating 7-dehydrocholesterol with ultraviolet B (UVB) radiation and, to a lesser extent, through dairy products such as milk, codfish, mushrooms, eggs, and fortified food, as well as vitamin D supplementation [3]. Serum 25-hydroxyvitamin D (25(OH)D) is a valid biomarker of vitamin D status. The vitamin D range is based on numerous factors and differs across populations. Despite the constant controversy surrounding vitamin D deficiency, the most widely agreed upon definition for adults is a serum 25(OH)D level of less than 20 ng/mL, insufficiency between 20 and 30 ng/mL, and sufficiency above 30 ng/mL [4].

Recent studies suggest that there is growing concern about the worldwide prevalence of vitamin D insufficiency and deficiency [5]. It is estimated that nearly one billion people around the globe are affected by inadequate levels of this vital vitamin. In particular, populations in Europe, the Middle East, China, and Japan are at increased risk [6]. Vitamin D insufficiency is a concern for women in the Middle East [7,8]. Vitamin D insufficiency in women in the Middle East is primarily attributed to cultural practices, such as clothing habits that cover most of the body, which limit sun exposure necessary for vitamin D synthesis. Despite the region's abundant sunshine, many women spend considerable time indoors due to social and cultural factors, reducing their exposure to sunlight. Additionally, seasonal variation and air pollution can also contribute to lower vitamin D levels.

The prevalence of vitamin D deficiency ranges widely, ranging from 18% to 84%, and is influenced by factors such as the country of residence and local cultural practices [9]. Additional contributors to vitamin D deficiency include insufficient sun exposure, lifestyle choices, darker skin tones, warm climates, and prolonged breastfeeding without the supplementation of calcium and vitamin D [10]. Deficiencies in vitamin D are also prevalent in Lebanon, although the country has a Mediterranean climate, with ample sunlight throughout the year [11]. Thus, it is important to study the level of knowledge, attitudes, and practices (KAP) regarding vitamin D among populations. Given these concerns, it is essential to assess how well young Lebanese adults understand vitamin D's role, sources, and health implications. Investigating their knowledge, attitudes, and practices will provide valuable insights into potential gaps and inform strategies for improving awareness and prevention efforts. The primary aim of this study is to assess the knowledge, attitudes, and practices (KAP) regarding vitamin D among the Lebanese population with emphasis on young adults.

## Materials and methods

Study design, sample size, and data collection

We used a quantitative cross-sectional design to investigate knowledge regarding vitamin D, along with attitudes and practices among the young adult population in Lebanon. The study was conducted between August 2023 and January 2024. Research data were collected through a self-administered online survey via Google Forms (Google, Inc., Mountain View, CA). The online questionnaire included an introduction that described the aim of the research and stated that participation was voluntary and anonymous and that the information collected via the survey would be used only for research purposes. Among the 370 responses, 16 were incomplete or invalid and were excluded, and 354 responses met the inclusion criteria.

Eligibility criteria included adult men or women aged 18-65 years residing in Lebanon. The data collection content was chosen and refined through a comprehensive analysis of previously published studies [12-14]. The questionnaire included four sections. The first part described the respondents' sociodemographic characteristics. The second section consisted of 12 queries related to vitamin D knowledge and an additional question about the source of that knowledge. The third part contained six questions exploring attitudes toward vitamin D, while the final part involved 18 practice-related questions concerning vitamin D. The questionnaire was provided in two languages, Arabic and English, to ensure accessibility.

Pilot study

We carried out a pilot study involving 16 participants to evaluate the survey before its full implementation. On the basis of these findings, adjustments were made to enhance clarity and comprehension. Data from the pilot study were excluded from the analysis presented hereafter. The survey's reliability was evaluated via a test-retest method, confirming its internal consistency. A value of 0.752 from Cronbach's alpha coefficient test demonstrated acceptable internal consistency.

Methodology for assessing knowledge and attitudes

For the knowledge assessment, scores were assigned on a scale of 0-40, with a mean cutoff of 21. The knowledge levels were categorized as poor for values below 20 and good for values equal to or exceeding 21. For the attitude evaluation, the participants who correctly answered four or more out of six questions were deemed to possess a positive attitude. Conversely, those with a score of 3 or less points were categorized as exhibiting a negative attitude.

Ethical approval

The Lebanese International University (LIU) Institutional Review Board approved the study under ethical authorization (approval number: LIUIRB-240208-SS-324). The participants in the research project were informed and provided agreement, with the understanding that their information would remain confidential.

Statistical analysis

The statistical analysis was conducted via the Statistical Package for Social Sciences (SPSS) version 26 (IBM SPSS Statistics, Armonk, NY) and Microsoft Office Excel 2016 (Microsoft Corp., Redmond, WA). The descriptive statistics present frequencies and percentages to show sociodemographic characteristics and responses regarding knowledge and attitudes toward vitamin D. The chi-square test was applied along with cross-tabulation analysis to assess correlations between sociodemographic characteristics and knowledge and attitudes toward vitamin D. A P value of ≤0.05 was used to indicate statistical significance.

## Results

The survey included 354 participants; 241 (68.1%) were women, and 113 (31.9%) were men. Among the total female percentage, 41.8% (148) are veiled. The majority of the participants (64.4%, 228) were between the ages of 18 and 23, whereas 75 (21.2%) were between the ages of 24 and 29. In terms of educational status, a significant proportion of respondents, specifically 58.8% (187), were pursuing bachelor's degrees. In terms of marital status, the majority of the participants (298, 84.2%) were unmarried, 204 (48.5%) were students, and 124 (29.5%) and 39 (9.3%) were employed indoors and outdoors, respectively. The majority of the participants (60.2%, 213) stated that their skin tones varied from fair/light to beige. A total of 88 (23.2%) individuals reported having a light-brown/olive skin tone, whereas eight (2.3%) reported having dark-brown skin. Table 1 presents a detailed description of the participants' demographic characteristics.

**Table 1:** Demographic characteristics of the participants

Description	N (%)	Knowledge Score	Attitude Score
Age (years)		P=0.239	P=0.583
18-23	228 (64.4%)		
24-29	75 (21.2%)		
30-35	30 (8.5%)		
36-41	12 (3.4%)		
42-47	7 (2.7%)		
48-53	2(0.6%)		
Gender		P=0.002	P=0.001
Male	113 (31.9%)		
Female	241 (68.1%)		
If you are a woman, do you wear a scarf?			
Yes	148 (41.8%)		
No	116 (32.8%)		
Marital status		P=0.118	P=0.173
Unmarried	298 (84.2%)		
Married	56 (15.8%)		
Education level		P=0.001	P=0.776
High school	55 (15.5%)		
Bachelor's degree	187 (58.8%)		
Diploma	36 (10.2%)		
Master's degree	31 (8.8%)		
PhD	5 (1.45%)		
Others	40 (11.3%)		
Occupation (multiple responses)		P=0.192	P=0.745
Indoor employed	124 (29.5%)		
Outdoor employed	39 (9.3%)		
Student	204 (48.5%)		
Retired	0 (0%)		
Unemployed	54 (12.8%)		
Skin color			
Fair or pale	51 (14.4%)		
Fair/light to beige	213 (60.2%)		
Light brown/olive	88 (23.2%)		
Dark brown	8 (2.3%)		

Most participants had sufficient knowledge about sources of vitamin D, primarily from the internet and physicians (17.6%, 175). Sunlight was recognized as the most crucial source. Additionally, 244 (33.7%) of the participants acknowledged the role of vitamin D in preventing osteoporosis, whereas 148 (20.4%) believed that it was aiding in calcium absorption. Significant levels of knowledge and positive attitudes were evident among more than 266 (75%) of the participants. However, over half expressed concerns about their vitamin D levels. Most were willing to take supplements (92.7%, 330) and undergo testing (92.1%, 326).

**Figure 1:**
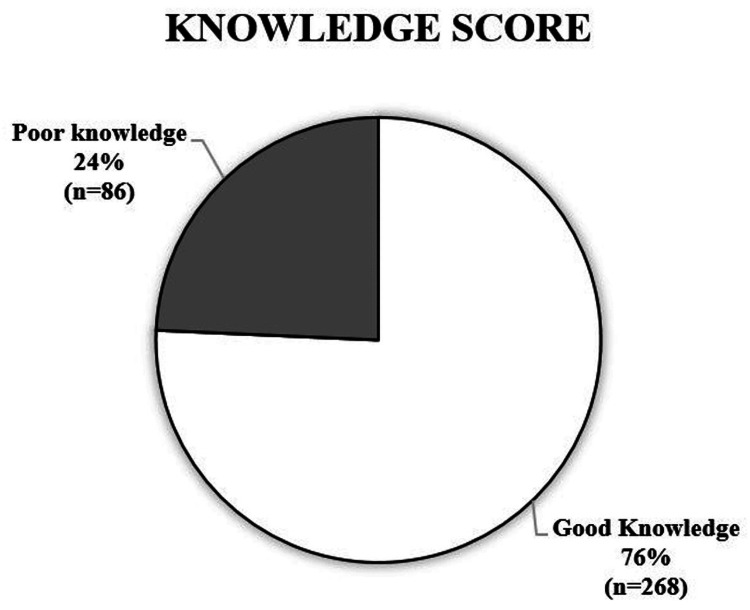
Participants' overall knowledge scores

A significant percentage of individuals (75.7%, 268) had adequate knowledge about vitamin D, while 86 (24.3%) had below-average levels (Figure 1).

**Figure 2:**
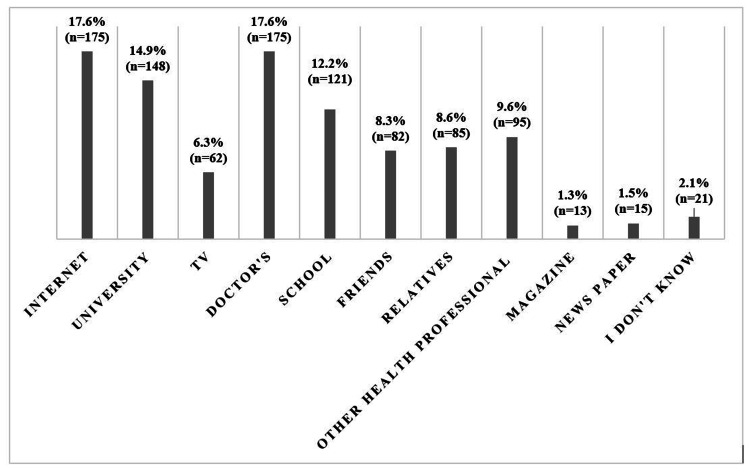
Knowledge sources of vitamin D

Our findings showed that education (chi-square=22.28; P value=0.001) and sex (chi-square=9.425; P value=0.002) had significant effects on vitamin D knowledge. Our results indicated that the internet and physicians accounted for 17.6% (175) of the primary knowledge sources, followed by colleges (14.9%, 148) and schools (12.2%, 121) (Figure 2).

Nearly all of those involved in the study knew that vitamin D is present in our body, and 198 (35.5%) of the participants were aware of its source. While 252 (71.2%) believed that meals alone could not provide sufficient vitamin D, the correct identification of dietary sources varied, with fatty fish being recognized by 181 (29.6%) and olive oil being recognized by only 43 (7.1%). Benefits, such as preventing osteoporosis (33.7%, 244) and assisting in calcium absorption (20.4%, 148) were reported. However, 142 (40.1%) of the respondents were aware of the correct range of vitamin D (20-50 ng/mL), whereas 167 (47.2%) were uncertain. With respect to the duration required for sun exposure to attain adequate vitamin D levels, 174 (49.2%) of the participants accurately reported the recommended duration of 10-20 minutes per day. Additionally, 211 (59.6%) were unaware of the recommended daily dosage of 600 IU. The causes of insufficiency were identified as inadequate meals (35.4%, 260) and insufficient sun exposure (38.6%, 283), with limited knowledge of additional factors. The awareness of the association of vitamin D with osteoporosis (32.4%, 281) and depression (18.7%, 162) varied, with only 31 (3.6%) of the respondents identifying cancer as a possible consequence of vitamin D deficiency. Table 2 shows the participants' knowledge of vitamin D.

**Table 2:** Survey questions and response percentages on knowledge, attitudes, and practices related to vitamin D SPF: sun protection factor

	Description	N (%)
Knowledge questions regarding vitamin D	Have you ever heard or learned about vitamin D?	
	Yes	328 (92.7%)
	No	26 (7.3%)
	Where did you hear or learn about vitamin D? (multiple responses)	
	Newspaper	15 (1.5%)
	Magazine	13 (1.3%)
	TV	62 (6.3%)
	Doctors	175 (17.6%)
	Friends	82 (8.3%)
	Relatives	85 (8.6%)
	School	121 (12.2%)
	University	148 (14.9%)
	Internet	175 (17.6%)
	Other health professionals	95 (9.6%)
	I do not know	21 (2.1%)
	Is vitamin D present in the body?	
	Yes	285 (80.5%)
	No	33 (9.3%)
	I do not know	36 (10.2%)
	Do you know the function and benefits of vitamin D? (multiple responses)	
	Prevents osteoporosis	244 (33.7%)
	Prevents general weakness	144 (19.9%)
	Prevents chronic disease	58 (8%)
	Prevents cancer	43 (5.9%)
	Aids in calcium absorption	148 (20.4%)
	Good for vision	43 (5.9%)
	I do not know	45 (6.2%)
	Where do you think the body gets vitamin D? (multiple responses)	
	Diet	61 (11%)
	Exposure to the sun	176 (31.6%)
	Food supplement	109 (19.6%)
	All the above	198 (35.5%)
	I do not know	13 (2.3%)
	How much time do you need to spend in the sun to get enough vitamin D?	
	<10 minutes	48 (13.6%)
	10-20 minutes	174 (49.2%)
	30-60 minutes	88 (24.9%)
	>1 hour	38 (10.7%)
	Missing	6 (1.7%)
	Meals alone can provide sufficient levels of vitamin D	
	Yes	45 (12.7%)
	No	252 (71.2%)
	I do not know	57 (16.1%)
	What type of food is a good source of vitamin D? (multiple responses)	
	Vegetables and fruits	95 (15.5%)
	Milk	121 (19.8%)
	Fatty fish: salmon	181 (29.6%)
	Olive oil	43 (7.1%)
	Eggs	99 (16.2%)
	I do not know	72 (11.8%)
	Aware of normal serum vitamin D levels	
	Yes	166 (46.9%)
	No	188 (53.1%)
	What is the normal level of vitamin D (ng mL^-1^)?	
	<12	13 (3.7%)
	<20	32 (9%)
	20-50	142 (40.1%)
	I do not know	167 (47.2%)
	What are the daily needs for vitamin D (IU)?	
	200 IU	39 (11%)
	600 IU	88 (24.9%)
	800 IU	16 (4.5%)
	I do not know	211 (59.6%)
	What are the causes of vitamin D deficiency? (multiple responses)	
	Hereditary	90 (12.3%)
	Less exposure to the sun	283 (38.6%)
	Lack of eating vitamin D-rich food	260 (35.4%)
	Kidney disease	49 (6.7%)
	Respiratory disease	8 (1.1%)
	I do not know	43 (5.9%)
	Complications for vitamin D deficiency (multiple responses)	
	Osteoporosis	281 (32.4%)
	Hair loss	212 (24.4%)
	Depression	162 (18.7%)
	Acne	66 (7.6%)
	Obesity	50 (5.8%)
	Cancer	31 (3.6%)
	Eczema	22 (2.5%)
	Blindness	20 (2.3%)
	Peptic ulcer	24 (2.7%)
Attitude questions regarding vitamin D	Vitamin D is vital for overall health	
	Yes	352 (99.4%)
	No	2 (0.6%)
	I like to expose myself to sunlight	
	Yes	292 (82.5%)
	No	62 (17.5%)
	The exposure to sunlight is harmful to the skin	
	Yes	207 (58.5%)
	No	147 (41.5%)
	I am concerned that my current vitamin D levels might be too low	
	Yes	201 (56.8%)
	No	153 (43.2%)
	Interested to know about vitamin D deficiency-associated symptoms	
	Yes	31.7 (89.5%)
	No	37 (10.5%)
	I am willing to undergo a test for vitamin D if a medical condition demands it	
	Yes	326 (92.1%)
	No	28 (7.9%)
Vitamin D-related practices	Have you ever examined your vitamin D level?	
	Yes	207 (58.5%)
	No	147 (41.5%)
	Do you take food supplements or a multivitamin containing vitamin D?	
	Yes	193 (54.5%)
	No	161 (45.5%)
	If no, what is the primary reason for not taking vitamin D?	
	Never thought about it	116 (32.8%)
	Feels getting enough vitamin D from diet	32 (9%)
	Feels getting enough vitamin D from the sun	23 (6.5%)
	I prefer not to take any supplements/vitamins	30 (8.5%)
	Missing	153 (43.2%)
	If yes, what is the vitamin D dose of the supplement?	
	<1000 IU	62 (17.5%)
	1000 IU and over	81 (22.9%)
	I do not know	144 (40.7%)
	Missing	67 (18.9%)
	Motivation for starting to take vitamin D	
	A doctor or health professional recommended	209 (59%)
	A friend or family member recommended	30 (8.5%)
	Read about vitamin D on the internet or in a book/magazine	42 (11.9%)
	Someone else in the family is taking it	32 (9%)
	Missing	41 (11.6%)
	Previously investigated vitamin D serum levels among family members	
	Yes	147 (41.5%)
	No	207 (58.5%)
	If yes, what was the result?	
	Normal vitamin D levels	27 (7.6%)
	Vitamin D deficiency	122 (34.5%)
	Excess vitamin D level	1 (0.3%)
	I do not know	91 (25.7%)
	Missing	113 (31.9%)
	How do you feel about sun exposure?	
	I like getting exposed to the sun all the time	56 (15.8%)
	I like getting exposed to the sun sometimes	220 (62.1%)
	I rarely get exposed to the sun	40 (11.3%)
	I avoid getting exposed to the sun	28 (7.9%)
	I do not know	10 (2.9%)
	What time do you prefer to be exposed to the sun?	
	Early morning	170 (47.8%)
	Afternoon	70 (19.7%)
	At noon	63 (17.7%)
	I avoid sunlight	24 (6.7%)
	I usually go out at night but not during the day	11 (3.1%)
	I do not know	16 (4.4%)
	Missing	2 (0.6%)
	Which season do you like to be exposed to the sun?	
	Summer	92 (25.8%)
	Autumn	20 (5.6%)
	Spring	93 (26.1%)
	Winter	70 (19.7%)
	All year	79 (22.2%)
	Missing	2 (0.6%)
	How frequently are you exposed to the sun? (times per week)	
	0-1	53 (14.6%)
	2-3	116 (32.6%)
	>3	138 (38.7%)
	I do not know	48 (13.5%)
	Missing	2 (0.6%)
	Duration of sun exposure	
	15 minutes or less	147 (41.3%)
	15-29 minutes	72 (20.2%)
	30-60 minutes	50 (14%)
	More than an hour	42 (11.8%)
	I do not know	45 (12.7%)
	What do you use most often as protection from the sun?	
	Umbrella	7 (2%)
	Sunscreen	205 (57.6%)
	Hat	35 (9.8%)
	Do not use any protection	101 (28.4%)
	I wear a scarf, and that is enough for me	6 (1.7%)
	Missing	2 (0.6%)
	Do you use sunscreen products that contain SPF>15?	
	Yes	195 (54.8%)
	No	115 (32.3%)
	I do not know	44 (12.3%)
	Missing	2 (0.6%)
	At what season do you prefer to use sunscreen?	
	Summer	173 (48.6%)
	Winter	5 (1.4%)
	Both	128 (36%)
	I do not know	48 (13.5%)
	Missing	2 (0.6%)
	How often do you use sunscreen?	
	Always	118 (33.1%)
	Sometimes	109 (30.6%)
	Rarely	74 (20.8%)
	Never used sunscreen	53 (14.9%)
	Missing	2 (0.6%)
	What practices do you apply to prevent vitamin D deficiency? (multiple responses)	
	Exposure to sunlight	213 (35.1%)
	Take a vitamin D supplement	188 (31%)
	Drink two cups of milk	52 (8.6%)
	Not using SPF-containing creams	18 (3%)
	Increase seafood in the diet	73 (12%)
	None	62 (10.2%)
	Willing to take vitamin D regularly if prescribed by physicians	
	Yes	330 (92.7%)
	No	24 (6.7%)
	Missing	2 (0.6%)

**Figure 3:**
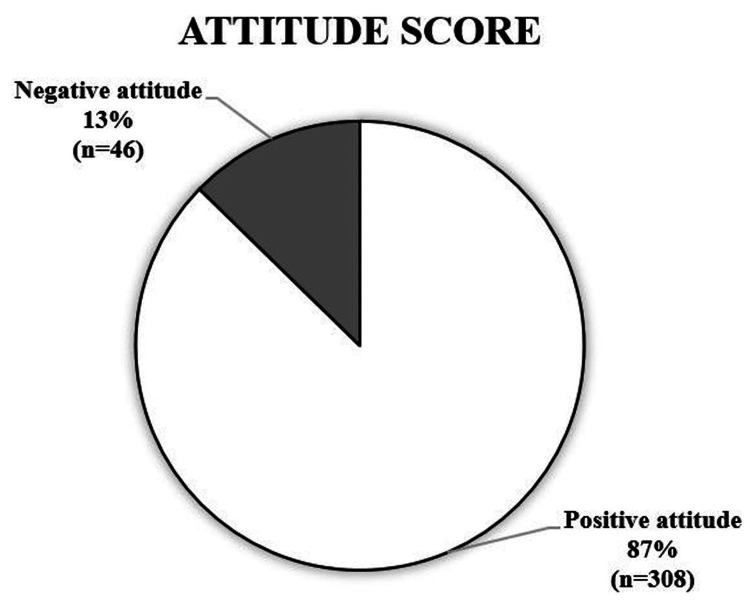
Participants' overall attitude scores

Our study revealed that the majority of the participants (87.3%, 309) had a positive attitude toward vitamin D, whereas 12.7% (45) had a negative attitude (Figure 3). Statistics revealed a significant association between attitudes and gender (chi-square=10.877; P value=0.001).

In addition, 352 (99.4%) of the participants acknowledged the vital role of vitamin D in health. Although 292 (82.5%) expressed a preference for sun exposure, more than half (58.5%, 207) believed that it could harm the skin. There was clear concern about vitamin D levels, with 201 (56.8%) of the respondents expressing concern and 153 (43.2%) being unconcerned. Furthermore, a significant majority (89.5% and 92.1%, respectively) expressed interest in learning about symptoms associated with vitamin D deficiency and were willing to undergo testing if it was recommended by a medical professional. Details regarding the attitudes toward vitamin D are provided in Table 2.

Among the study participants, 207 (58.5%) reported having vitamin D serum levels tested, whereas 193 (54.5%) indicated that they were taking food supplements or multivitamins containing vitamin D. Of those individuals, 81 (22.9%) consumed 1000 IU or more of vitamin D, while 62 (17.5%) consumed less than 1000 IU. The majority (92.7%, 330) were willing to take vitamin D if recommended by physicians. When questioned about their preferences regarding sun exposure, 220 (62.1%) preferred exposure to the sun sometimes, whereas 28 (7.9%) avoided it altogether. Among those who indicated that they tended to spend time in the sun, 170 (47.8%) preferred morning hour exposure, followed by 70 (19.7%) in the afternoon. In terms of direct sun exposure frequency, 138 (38.7%) stated that they spent more than three times per week in direct sunlight. Moreover, 205 (57.6%) used sunscreen, primarily with an sun protection factor (SPF)>15, and the frequency of its application varied. The frequency of sunscreen application was reported to be 33.1% (118). With respect to practices to prevent vitamin D deficiency, sunlight exposure (35.1%, 213) and the use of vitamin D supplements (31%, 188) were included. Table 2 describes vitamin D-related practices.

**Figure 4:**
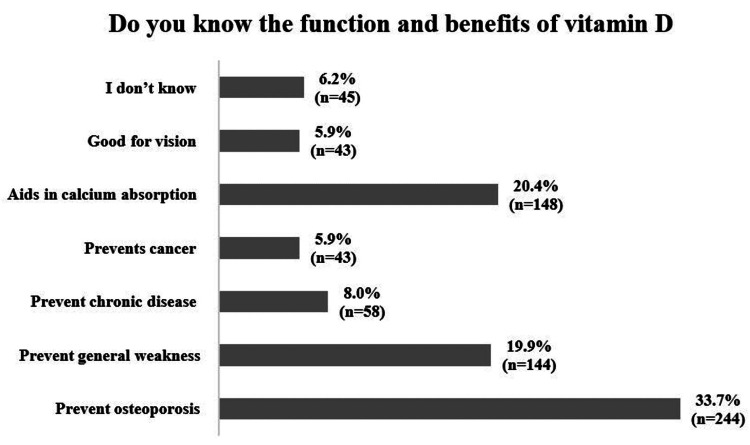
Functions and benefits of vitamin D

In response to the question on the functions and benefits of vitamin D, the most recognized benefit among the participants was its role in preventing osteoporosis (33.7%, 244), followed by aiding in calcium absorption (20.4%, 148) and preventing general weakness (19.9%, 144). Fewer participants associated vitamin D with preventing chronic diseases (8%, 58) and cancer (5.9%, 43), while an equal number (5.9%, 43) linked it to vision health. Notably, 6.2% (45) of the respondents indicated that they were unaware of vitamin D's functions and benefits. These findings, summarized in Figure 4, highlight varying levels of awareness, with a stronger association between vitamin D and bone health compared to its broader physiological roles.

## Discussion

Our cross-sectional study, which aimed to evaluate the levels of knowledge, attitudes, and practices regarding vitamin D deficiency among the young population in Lebanon, is one of the very few studies in a country marked with significant deficiency in vitamin D [11]. The study of the participants' vitamin D knowledge, attitudes, and practices provides information about essential variables affecting public health. Understanding these characteristics provides useful insights into alternative interventions aimed at addressing deficiencies and promoting optimal vitamin D status. The findings revealed a good level of knowledge about vitamin D among the public, which aligns with similar results reported in other studies [13,15,16] and contradicts those of other studies [17,18]. Variations in vitamin D knowledge can be associated with various factors, including public health education, healthcare infrastructure, culture, dietary practices, and access to information. Moreover, economic differences, literacy levels, and healthcare accessibility play major roles in shaping knowledge about vitamin D. Countries with well-established healthcare systems and comprehensive health promotion efforts tend to exhibit greater awareness of vitamin D than countries with less developed healthcare systems and limited health education initiatives. The internet emerged as the primary source of information, similar to the findings of a previous study [12,13]; additionally, physicians played a significant role in increasing health awareness among the Lebanese, which contrasts with findings from Kuwait [19]. In accordance with findings from other studies, the participants presented satisfactory knowledge regarding the three primary sources of vitamin D: sunlight, food, and supplements [20-22].

Even though the participants demonstrated good knowledge regarding the role of the sun in providing vitamin D, almost half of the respondents were able to specify the required duration of sun exposure accurately to acquire enough vitamin D (10-20 minutes) [16]. This differs from a study conducted in Oman [23]. Most respondents correctly identified fatty fish (salmon) as the main dietary source of vitamin D, in line with previous research [24]. However, similar to our results, 95 (15.5%) individuals incorrectly stated that vegetables and fruits are sources of vitamin D [24]. According to Matsui (2020), a daily vitamin D dosage of 600 IU is recommended [16]. Our analysis demonstrated that only 22.9% of the participants were aware of this recommended dose, which aligns with the results reported in another study in Jordan [13]. Furthermore, approximately half of the participants indicated awareness of normal serum vitamin D levels, with two-fifths providing the correct answer. This contrasts with findings from other research conducted in Saudi Arabia [25]. Research carried out by Uzrail et al. (2021) revealed that the respondents knew that insufficient sunlight and a lack of vitamin D-rich foods cause vitamin D deficiency [13]. Only a few thought it could be hereditary [13]. Our investigation provides comparable findings. In addition, most participants were familiar with the benefits of vitamin D (Figure 4) in maintaining bone health by preventing osteoporosis and assisting in calcium absorption. However, our results revealed a low level of awareness regarding other benefits of vitamin D, such as its role in preventing cancer. Matching results were observed in other studies carried out in Jordan and Oman [13,23].

This research investigated attitudes and practices of young Lebanese adults toward vitamin D. The results revealed that nearly all the participants presented positive attitudes toward vitamin D. These findings contrast with those of Al Bathi et al. (2012) [19] in Kuwait but align with those of Alkalash et al. (2023) [12], where all the participants agreed on the importance of vitamin D for health. Additionally, more than half of the participants were concerned about sunlight exposure, and most were interested in learning about vitamin D deficiency symptoms and being tested if needed [12]. In terms of practices, our results diverge from those reported in a study in Kuwait, indicating a different pattern of behaviors [19]. The majority of the participants reported spending only 15 minutes or between 15 and 29 minutes per day in the sun, particularly during the early morning, which is in accordance with the findings of research conducted in Jordan [13]. Most of the respondents in our study expressed a preference for sun exposure during the spring and summer seasons, suggesting that they obtain adequate vitamin D from sunlight. This aligns with the idea that exposure during these seasons promotes the synthesis of vitamin D through the skin, as suggested by Hoge et al. (2015) [26]. Furthermore, our findings show that practices such as sun exposure and the use of vitamin D supplements to prevent vitamin D deficiency are similar to those reported in other studies [13].

Two-fifths of the family respondents examined their vitamin D levels; however, one-third of them were vitamin D deficient [11,27]. In addition, 34.8% of the 148 female participants who wore scarves consumed food supplements or multivitamins containing vitamin D. This behavior may be due to cultural and religious practices, where women are expected to cover their hair and bodies, which might lead to vitamin D deficiency. In addition, over half of the participants utilized sunscreen as a protective measure against the sun. Limited exposure to sunlight and increased sunscreen usage, particularly among women, have been identified as key factors contributing to vitamin D deficiency, especially in women [28]. A considerable number of participants who use vitamin D supplements do so on the basis of recommendations from healthcare professionals or physicians; this demonstrates the importance of professional healthcare guidance in maintaining their health and overall well-being. In addition, 34.7% of the individuals who consumed food supplements or multivitamins containing vitamin D had fair-to-light beige skin tones. Importantly, individuals with darker skin tones are more prone to vitamin D deficiency. This is due to the presence of melanin, the pigment responsible for skin color, which limits the production of vitamin D3 in the skin. Therefore, individuals with darker skin need more sunlight exposure to generate sufficient vitamin D [29]. However, individuals with fair-to-light beige or pale skin tones may use more vitamin D tablets or multivitamins containing vitamin D since individuals with fair skin tones are more prone to sunburn and should therefore avoid prolonged sun exposure [30].

Limitations

The study of vitamin D knowledge, attitudes, and practices has limitations, including a sample that may not fully represent the population's diversity of knowledge and practices. Moreover, the predominance of female respondents in our study poses a potential limitation. Additionally, the use of an online survey introduces the possibility of self-selection bias, where health-conscious individuals may be overrepresented.

## Conclusions

The study examines the knowledge, attitudes, and practices of the young Lebanese population regarding vitamin D. This study reveals that people are generally well-informed about its sources and benefits, despite some misconceptions. Most people are interested in learning about vitamin D deficiency symptoms and testing if necessary. However, there is potential for improvement in sun exposure practices and vitamin D supplementation adherence among specific demographic groups. The findings can be used to improve targeted interventions and public health campaigns in Lebanon.

## References

[1] Khazai N, Judd SE, Tangpricha V (2008). Calcium and vitamin D: skeletal and extraskeletal health. Curr Rheumatol Rep.

[2] Nagaria TD, Shinde RK, Shukla S, Acharya S, Acharya N, Jogdand SD (2023). The sunlight-vitamin D connection: implications for patient outcomes in the surgical intensive care unit. Cureus.

[3] Tabrizi R, Moosazadeh M, Akbari M (2018). High prevalence of vitamin D deficiency among Iranian population: a systematic review and meta-analysis. Iran J Med Sci.

[4] Holick MF (2007). Vitamin D deficiency. N Engl J Med.

[5] Mithal A, Wahl DA, Bonjour JP (2009). Global vitamin D status and determinants of hypovitaminosis D. Osteoporos Int.

[6] Vieth R, Bischoff-Ferrari H, Boucher BJ (2007). The urgent need to recommend an intake of vitamin D that is effective. Am J Clin Nutr.

[7] Palacios C, Gonzalez L (2014). Is vitamin D deficiency a major global public health problem?. J Steroid Biochem Mol Biol.

[8] Hussain AN, Alkhenizan AH, El Shaker M, Raef H, Gabr A (2014). Increasing trends and significance of hypovitaminosis D: a population-based study in the Kingdom of Saudi Arabia. Arch Osteoporos.

[9] Dawodu A, Wagner CL (2007). Mother-child vitamin D deficiency: an international perspective. Arch Dis Child.

[10] El Rifai NM, Abdel Moety GA, Gaafar HM, Hamed DA (2014). Vitamin D deficiency in Egyptian mothers and their neonates and possible related factors. J Matern Fetal Neonatal Med.

[11] Arabi A, Chamoun N, Nasrallah MP, Tamim HM (2021). Vitamin D deficiency in Lebanese adults: prevalence and predictors from a cross-sectional community-based study. Int J Endocrinol.

[12] Alkalash SH, Odah M, Alkenani HH, Hibili NH, Al-Essa RS, Almowallad RT, Aldabali S (2023). Public knowledge, attitude, and practice toward vitamin D deficiency in Al-Qunfudhah Governorate, Saudi Arabia. Cureus.

[13] Uzrail A, Abu Assab M, Alkalbani R, Al Kofahi R, Kadhim A (2021). Knowledge, attitude and practice (KAP) towards vitamin D deficiency in the Jordanian adult population: a cross-sectional study. Res J Med Sci.

[14] Arora H, Dixit V, Srivastava N (2016). Evaluation of knowledge, practices of vitamin D and attitude toward sunlight among Indian students. Evaluation.

[15] Roth DE, Abrams SA, Aloia J (2018). Global prevalence and disease burden of vitamin D deficiency: a roadmap for action in low- and middle-income countries. Ann N Y Acad Sci.

[16] Matsui MS (2020). Vitamin D update. Curr Dermatol Rep.

[17] Aljefree N, Lee P, Ahmed F (2017). Exploring knowledge and attitudes about vitamin D among adults in Saudi Arabia: a qualitative study. Healthcare (Basel).

[18] Deschasaux M, Souberbielle JC, Partula V (2016). What do people know and believe about vitamin D?. Nutrients.

[19] Al Bathi BA, Al Zayed KE, Al Qenai M, Makboul G, El-Shazly MK (2012). Knowledge, attitude and practice of patients attending primary care centers toward vitamin D in Kuwait. Alex J Med.

[20] Boland S, Irwin JD, Johnson AM (2015). A survey of university students' vitamin D-related knowledge. J Nutr Educ Behav.

[21] Vu LH, van der Pols JC, Whiteman DC, Kimlin MG, Neale RE (2010). Knowledge and attitudes about vitamin D and impact on sun protection practices among urban office workers in Brisbane, Australia. Cancer Epidemiol Biomarkers Prev.

[22] Kung AW, Lee KK (2006). Knowledge of vitamin D and perceptions and attitudes toward sunlight among Chinese middle-aged and elderly women: a population survey in Hong Kong. BMC Public Health.

[23] Khan N, Hussain S, Bashar S, Hasan S, Palis E, Iqbal S (2017). Attitudes and behavior towards sunlight exposure and knowledge about vitamin D among Omani female university students. EC Nutr.

[24] O'Connor C, Glatt D, White L, Revuelta Iniesta R (2018). Knowledge, attitudes and perceptions towards vitamin D in a UK adult population: a cross-sectional study. Int J Environ Res Public Health.

[25] Kambal N, Abdelwahab S, Albasheer O (2023). Vitamin D knowledge, awareness and practices of female students in the southwest of Saudi Arabia: a cross-sectional study. Medicine (Baltimore).

[26] Hoge A, Donneau AF, Streel S (2015). Vitamin D deficiency is common among adults in Wallonia (Belgium, 51°30' North): findings from the Nutrition, Environment and Cardio-Vascular Health study. Nutr Res.

[27] Harkous D, Ghorayeb N, Gannagé-Yared MH (2023). Prevalence and predictors of vitamin D deficiency in Lebanon: 2016-2022, before and during the COVID-19 outbreak. Endocrine.

[28] AlGhamdi KM, AlAklabi AS, AlQahtani AZ (2016). Knowledge, attitudes and practices of the general public toward sun exposure and protection: a national survey in Saudi Arabia. Saudi Pharm J.

[29] Carlberg C (2022). Vitamin D and pigmented skin. Nutrients.

[30] D'Orazio J, Jarrett S, Amaro-Ortiz A, Scott T (2013). UV radiation and the skin. Int J Mol Sci.

